# Comparative Density Functional Theory Insights Into B_16_C_16_ and Si_16_C_16_ Nanocages for Sensing Oil‐Derived Fault Gases in Energy and Industrial Systems

**DOI:** 10.1002/open.70245

**Published:** 2026-06-16

**Authors:** Khalid Abdullah Alrashidi, Hafiz Ali Rizwan, Muhammad Usman Khan, Amir Sohail

**Affiliations:** ^1^ Department of Chemistry College of Science King Saud University Riyadh Saudi Arabia; ^2^ Department of Chemistry University of Okara Okara Pakistan; ^3^ Department of Chemistry College of Science United Arab Emirates University Al Ain UAE

**Keywords:** boron carbide nanocage, density functional theory, oil decomposed toxic gases, quantum theory of atoms in molecules, silicon carbide nanocage

## Abstract

Oil‐immersed electrical equipment, including power transformers and underground cables, generates fault‐related decomposition gases such as C_2_H_4_, CO, and H_2_S under abnormal operating conditions. Prompt and accurate detection of these toxic gases is critical for safe operations and system reliability. In this study, the adsorption and sensing behavior of these gases on boron carbide (B_16_C_16_) and silicon carbide (Si_16_C_16_) nanocages were investigated using density functional theory (DFT) at the B3LYP‐D3/6‐31G(d, p) level. Results show that gas adsorption is more effective on the BC nanocage than on SiC. The highest negative adsorption energies were recorded for C_2_H_4_ and CO on BC, reaching −80.274 and −84.580 kcal, for the C_2_H_4__C4_BC and CO_C6_BC systems, respectively. H_2_S adsorption on BC produced the lowest energy gaps of 1.867 and 1.914 eV in H_2_S_C6_BC and H_2_S_C4_BC, respectively, significantly enhancing electrical conductivity to 5.45 × 10^12^ S/m and 5.40 × 10^12^ S/m. BC‐based systems consistently yielded positive sensing responses, while SiC‐based systems showed mostly negative values. NCI and QTAIM analyses confirmed covalent and partially covalent interactions between the analytes and nanocages. These findings establish BC nanocages as superior candidates for detecting fault gases, offering theoretical guidance for next‐generation sensors in power transformer diagnostics, renewable energy safety monitoring, and industrial hazard detection.

## Graphical abstract

B_16_C_16_ and Si_16_C_16_ nanocages were investigated via DFT for sensing transformer oil fault gases (C_2_H_4_, CO, H_2_S). BC nanocages exhibit stronger adsorption, reduced energy gaps, enhanced electrical conductivity, and positive sensing responses compared to SiC. NCI and QTAIM analyses reveal covalent and partially covalent interactions. These results highlight BC nanocages as promising candidates for high‐performance gas sensors in energy and industrial systems.

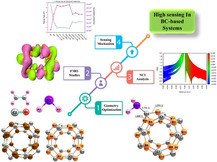

## 1 Introduction

Energy storage, gas separation, drug carriers, and many other uses of carbon‐based nanostructures have been proposed in recent decades for a wide range of industries and biological systems [1, 2, 3]. Molecular and atomic substances can interact with nanostructures due to their high surface‐to‐volume ratio [4, 5]. Fullerene, nanotubes, nanoparticles, nanophotonic, and graphene are only a few examples of carbon‐based nanostructures that have been developed so far using computational and experimental means [6, 7]. Because of their spherical form, fullerene and fullerene‐like nanostructures may be helpful in adsorption applications [8, 9, 10]. There have been many efforts to synthesize, characterize, and comprehend the nanoscale materials with such a large surface area since the invention of carbon nanotubes (CNTs) in 1991 [11, 12, 13, 14]. Materials on the nanoscale have the potential to be adsorbents for a variety of different chemicals due to their high surface‐to‐volume ratio. Materials such as nanoparticles, quantum dots, nanorods, nanowires, thin films, and even certain bulk materials may be classified as nanostructures based on their nanoscale dimensions [15, 16, 17]. Because of their unique characteristics, carbon nanostructures like graphene, fullerene, and carbon nanotubes (CNTs) have long piqued the interest of scientists [2, 18]. These materials have a wide range of potential uses, but they are most notable for their ability to remove contaminants from various environmental applications [19, 20]. It is crucial to consider ways to eliminate toxins from the living environment to maintain high standards of human health [21].

A lot of research has been conducted to develop novel adsorbents, especially those based on nanostructures, to detect and remove these types of pollutants [22, 23, 24, 25]. The physicochemical characteristics of fullerenes make them an attractive adsorbent for metal ions and a wide range of organic contaminants [26, 27, 28]. Chemical modifications might readily enhance the adsorption capacity and function of fullerenes [29, 30]. Boron carbide (BC) and silicon carbide (SiC) fullerene‐like materials have been previously reported for their oxidation resistance, high thermal conductivity, low dielectric constant, and high‐temperature stability, all of which are advantageous in a variety of electronics‐related applications [6, 31, 32]. Subsequently, a variety of nanomaterials, including graphene, organic frameworks (metal and covalent organic frameworks, among others), and electrochemical sensors for drug delivery have been produced [33, 34, 35, 36]. The proper functioning of an oil‐immersed power transformer directly impacts the reliability and security of the power system, making it a crucial component of any electrical transmission system. Partial discharge defects, which cause the transformer to overheat or partially discharge its insulating oil, become inevitable during prolonged operation and under harsh operating conditions [37, 38]. When transformers break down inside, the oil breaks down into several different gases, including carbon monoxide, hydrocarbons, methyl chloride, and ethane [39]. It follows that detecting these dissolved gases is an effective way to diagnose the state of an oil‐immersed power transformer and prevent faults [40]. This study will explore the complex world of nanocages with a view to adsorbing CO, C_2_H_4_, and H_2_S gas molecules. Also, one of the most significant atmospheric pollutants is carbon monoxide (CO), which is produced when gasoline is not burned completely [41]. Nearly five parts per million of carbon monoxide in the air is known to cause significant health problems and even death [42].

The future of nanomaterials in gas pollution detection and sensing is bright, according to a comprehensive review of academic works. The promise and versatility of structural engineering of nanomaterials for improved activities have been bolstered by a large body of research [43, 44]. According to research published by Deji and colleagues, pure graphene does not effectively absorb CO molecules. The adsorption energy increased when osmium was doped into graphene. Their research led them to the conclusion that CO sensors made of both‐edge osmium‐doped materials might be helpful in the future [45]. Additionally, Wang et al. found that Cu‐AD‐SA exhibited comparatively significant C_2_H_2_ adsorption in their paper [46]. Adsorption on the Fe_3_O_4_@Ag‐MOF nanocomposite has been the subject of more research. The Fe_3_O_4_@Ag‐MOF nanocomposite was shown to be an effective adsorbent for DOX and wastewater treatment, according to El‐Metwaly et al. [47].

Thus, this work aims to contribute to the literature by conducting an extensive computational investigation into the complex possibilities of BC and SiC nanocages. The first goal of this research is to develop state‐of‐the‐art sensors that selectively and sensitively detect CO, C_2_H_4_, and H_2_S molecules. The second goal is to learn everything we can about the gas adsorption mechanisms, including how surfaces are stabilized and the nature of interactions between them. In this study, we used a technique that encompasses a wide range of scientific inquiry by including a computational approach. The investigation begins with a geometric optimization to uncover the most stable geometric arrangements and the structural features of the adsorbent materials that are being researched. This prepares the groundwork for further investigations. Some of the methods included in these studies, are density of state (DOS), frontier molecular orbital (FMO), natural bond orbital (NBO), sensor mechanisms, reduced density gradient (RDG), and quantum theory of atoms in molecules (QTAIM).

## 2 Computational Methods

A computational model, B3LYP‐D3, was used for all density functional theory (DFT) computations to detect CO, H_2_S, and C_2_H_4_ gas molecules on the surfaces of B_16_C_16_ (BC) and Si_16_C_16_ (SiC) nanocages. B3LYP‐D3 is used in this study because it is a tried‐and‐true method for studying long‐range interactions in various nanostructures [48, 49]. Geometric optimization was carried out by a DFT calculation at 6‐31G(d, p) basis set [50] on the Gaussian 09 program [51], utilizing the B3LYP‐D3 functional. The geometry optimizations were performed using Gaussian 09’s default convergence criteria, where the maximum force, root mean square (RMS) force, maximum displacement, and RMS displacement were converged to thresholds of 0.000450, 0.000300, 0.001800, and 0.001200 a.u., respectively. The numerical integration was performed using the default Grid = Fine option in Gaussian 09, consisting of 75 radial shells and 302 angular points per shell, which has been well‐validated for accurate DFT calculations of molecular systems [52]. Using the graphical interface of the software Gauss View 5.0 [53], we drew and visualized all molecular structures before optimization. The optimization process led to the calculation of structural parameters, including adsorption energy (*E*_ads_), dipole moment, HOMO and LUMO energies, energy gap (*E*_g_), and quantum descriptors. The following Equation (1) was used to determine the adsorption energy of the complexes that were produced during the adsorption process:

(1) $$E_{\mathrm{ads}}=E_{\text{complex}}-\left(E_{\text{nanocage}}+E_{\mathrm{gas}}\right)+\mathrm{BSSE}$$

where *E*_complex_ is the overall energy of the adsorption complex and *E*_nanocage_ is the energy of each nanocage. We have *E*_gas_ for the gas molecules’ energy and *E*_ads_ for the resultant complexes’ adsorption energy. The density of state (DOS) plots for all complexes were created using the Multiwfn 3.8 program [54], and the data was subsequently displayed using the Origin software [55]. The visual molecular dynamics (VMD) software [56] was used to do HOMO–LUMO plots for designed systems. Using the Equations (2)–(6), we have determined the HOMO–LUMO energy gap and other quantum descriptors such as chemical softness (*σ*), chemical hardness (*η*), chemical potential (*μ*), and electrophilicity index (*ω*).

(2) $$E_{g}=E_{\mathrm{LUMO}}-E_{\mathrm{HOMO}}$$

(3) $$\sigma =\frac{1}{2\eta }=\frac{1}{\mathrm{IP}-\mathrm{EA}}$$

(4) $$\eta =\frac{1}{2}\;\left(\mathrm{IP}-\mathrm{EA}\right)=\frac{E_{\mathrm{LUMO}}-E_{\mathrm{HOMO}}}{2}$$

(5) $$\mu =-\frac{\left(I+A\right)}{2}$$

(6) $$\omega =\frac{\mu^{2}}{2\eta }$$

Natural bond orbital (NBO) studies were carried out to confirm the nature of charge transfer between the nanocages and adsorbed gases. Noncovalent interactions (NCI) and quantum theory of atoms in molecules (QTAIM) were used to study the nature of interactions and bond types forming between the two surfaces. In the end, various parameters of the sensing mechanism, like electrical conductivity, work function, and recovery time, were explored to better understand the sensing performance of the proposed nanocages.

## 3 Results and Discussion

### 3.1 Optimization of Geometry

**FIGURE 1:**
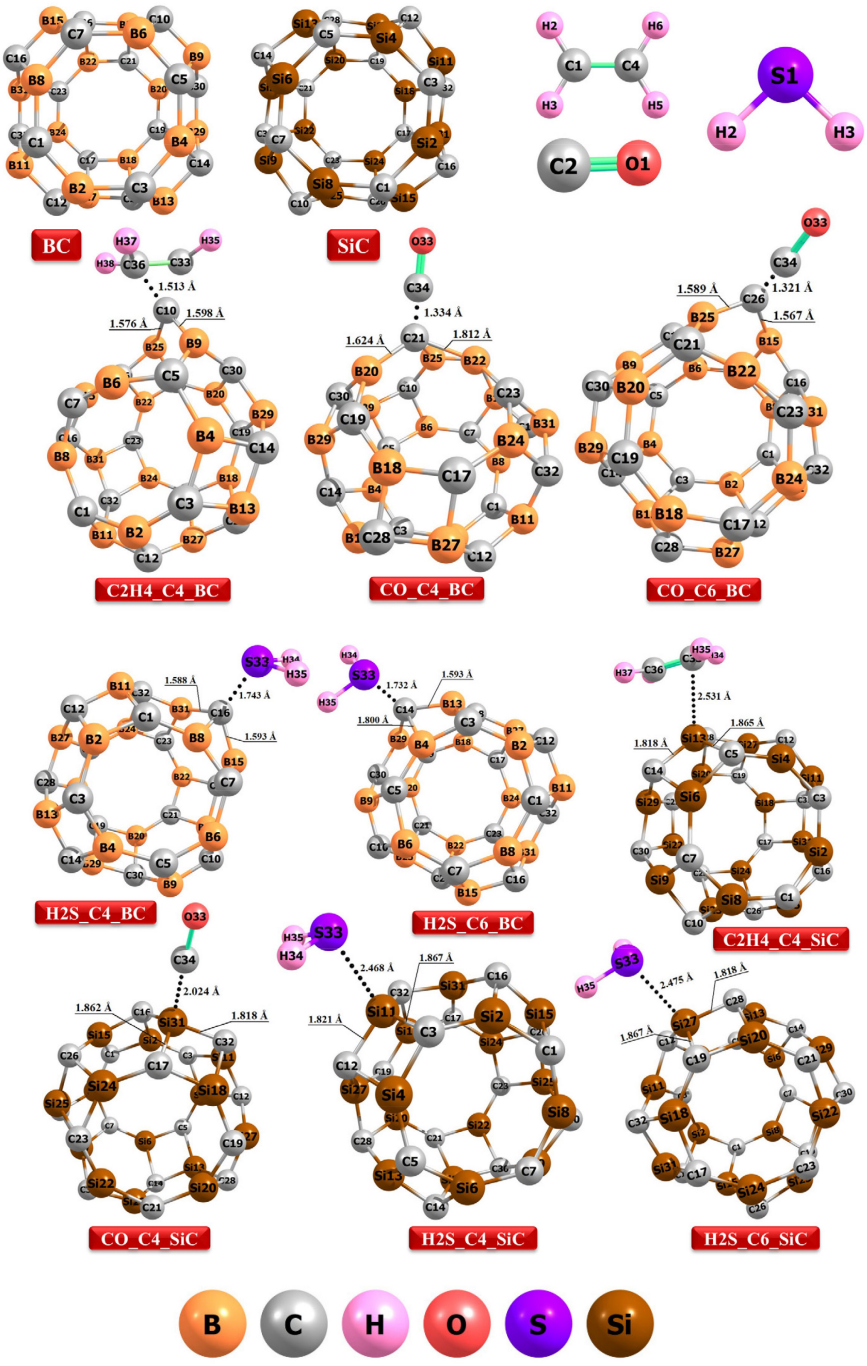
Optimized geometries of C_2_H_4_, CO, H_2_S, BC, and SiC before adsorption and all the developed systems after the adsorption process.

The optimal adsorption configuration was obtained by first ensuring stable conformance of the geometric structures via geometric optimization and then performing energy minimization. Three distinct optimized structures of ethene (C_2_H_4_), hydrogen sulfide (H_2_S) and carbon monoxide (CO) gases were achieved. Before the adsorption process, the naked BC and SiC were optimized independently. The optimized geometries of gases and nanocages are visualized in Figure 1. The optimized CO gas molecule’s carbon–oxygen bond length was determined to be 1.126 Å, which agrees with the results of the earlier theoretical calculation [57, 58]. In addition, the C_2_H_4_ gas molecule had carbon–carbon bond lengths of 1.326 Å and carbon–hydrogen bond lengths of 1.085 Å. The findings are in line with those of earlier computational investigations [59].

The adsorption of C_2_H_4_, CO, and H_2_S gas molecules results in the formation of optimized complex structures, as shown in Figure 1. Table 1 summarizes the surface bond lengths before and after adsorption. According to Table 1, the adsorption of toxic gases results in an increase in the bond lengths of the BC and SiC surfaces. Adsorption induces elongation of surface bonds. For example, after the adsorption of C_2_H_4_ on the BC surface, the bond length of the B_9_─C_10_ bond increases from 1.539 to 1.598 Å. Similarly, the adsorption of ethene on SiC nanocage increased the bond length of the C_5_─Si_13_ bond from 1.847 to 1.865 Å. CO adsorption on BC nanocage increased its bond length from 1.553 to 1.812 Å for the C_21_─B_22_ bond. Adsorption of CO on SiC nanocage also elevates its bond length from 1.802 to 1.818 Å for the C_32_─Si_31_ bond. Similarly, the bond lengths of C_16_─B_15_ and C_16_─B_31_ increased from 1.543 and 1.550–1.593 and 1.588 Å after the adsorption of H_2_S gas on BC nanocage. Adsorption of hydrogen sulfide on SiC increases its bond length from 1.802 and 1.847–1.821 and 1.867 Å for Si_11_─C_12_ and Si_11_─C_3_, respectively. This increase in bond lengths after adsorption is due to changes in the electronic clouds of the designed systems. Significant charge transfer may occur between toxic gases and nanocages, leading to variations in bond lengths.

**TABLE 1:** The computed bond lengths of designed systems before and after the adsorption of toxic gases onto BC and SiC nanocages.

Systems	Bond label	Bond lengths, Å	
		Before	After
C_2_H_4__C4_BC	B_9_─C_10_	1.539	1.598
	B_25_─C_10_	1.568	1.576
CO_C4_BC	C_21_─B_22_	1.553	1.812
	C_21_─B_20_	1.575	1.624
CO_C6_BC	B_15_─C_26_	1.559	1.567
	B_25_─C_26_	1.54	1.589
H_2_S_C4_BC	C_16_─B_15_	1.543	1.593
	C_16_─B_31_	1.550	1.588
H_2_S_C6_BC	B_13_─C_14_	1.543	1.593
	C_14_─B_4_	1.576	1.800
C_2_H_4__C4_SiC	C_5_─Si_13_	1.847	1.865
	Si_13_─C_14_	1.802	1.818
CO_C4_SiC	C_32_─Si_31_	1.802	1.818
	Si_31_─C_17_	1.847	1.862
H_2_S_C4_SiC	Si_11_─C_12_	1.802	1.821
	Si_11_─C_3_	1.847	1.867
H_2_S_C6_SiC	C_28_─Si_27_	1.802	1.818
	Si_27_─C_19_	1.847	1.867

### 3.2 Adsorption Energy and Dipole Moment

Adsorption energy (*E*_ads_) measures how efficiently an adsorbent takes in a gas or adsorbate molecule. Here, we will use the adsorption energy values to study the adsorption of C_2_H_4_, CO, and H_2_S gas molecules onto BC and SiC nanocages. Once the optimal adsorption configuration was determined, the *E*_ads_ calculation was performed using Equation (1). In addition, the findings for adsorption energy estimates and other important metrics, such as basis set superposition error (BSSE) and dipole moment, are summarized in Table 2. It should be noted that chemisorption is characterized by a negative adsorption energy value and physisorption by a positive value [60]. Crucially, adsorption strength is proportional to the negative magnitude of the adsorption energy [61]. Strong adsorption is related to the high negative adsorption energy with short interaction distance. Increasing the interaction distance results in lower adsorption energy of designed systems. The adsorption of C_2_H_4_, CO, and H_2_S gas molecules on the BC nanocage results in higher adsorption energies and shorter interaction distances. The highest adsorption energy is computed for the CO_C6_BC system, with −84.580 kcal/mol at an interaction distance of 1.321 Å. The C_2_H_4__C4_BC has the second‐best adsorption energy, calculated at −80.274 kcal, with an interaction distance of 1.513 Å. The weak adsorption among boron carbide (BC) systems is calculated for H_2_S_C4_BC, having an adsorption energy of −23.117 kcal at the interaction distance of 1.743 Å. The adsorption energies of SiC systems are lower than those of BC systems because of their long interaction distances. Among SiC systems, the highest value of adsorption energy is −13.144 kcal, which is calculated for H_2_S_C4_SiC with an interaction distance of 2.468 Å. The lowest adsorption energy among the SiC systems is calculated for CO_C4_SiC, with a value of −8.959 kcal at an interaction distance of 2.024 Å. The chemisorptive nature of interactions is persistent in both BC and SiC systems, but adsorption is stronger in BC systems due to their shorter interaction distance. Moreover, dipole moment values are calculated to assess charge separation between the two surfaces. High dipole moment values are responsible for better charge transfer and high sensing response. The dipole moments of BC and SiC before adsorption were 0.013 and 0.002 D, respectively, and both increased. The highest value of dipole moment among BC systems is calculated for H_2_S_C4_BC which has a dipole moment of 7.127 D. H_2_S_C4_SiC has the highest value of dipole moment among SiC systems having value of 6.304 D.

**TABLE 2:** The calculated adsorption energy values, basis set superposition error (BSSE), interaction distance, charge transfer, and dipole moment for designed systems.

Systems	Adsorption energy (*E*_ads_)	BSSE	Distance of interaction (*D*_int_)	Charge transfer (*Q*_t_)	Dipole moment
BC	—	—	—	—	0.013
SiC	—	—	—	—	0.002
C_2_H_4__C4_BC	−80.274	5.240	1.513	−0.031	0.802
CO_C4_BC	−55.661	4.025	1.334	0.092	1.480
CO_C6_BC	−84.580	4.129	1.321	0.015	1.119
H_2_S_C4_BC	−23.117	2.798	1.743	0.530	7.127
H_2_S_C6_BC	−24.802	2.832	1.732	0.499	6.649
C_2_H_4__C4_SiC	−9.524	2.880	2.531	0.206	5.130
CO_C4_SiC	−8.959	3.778	2.024	0.174	3.256
H_2_S_C4_SiC	−13.144	1.814	2.468	0.279	6.304
H_2_S_C6_SiC	−12.698	1.972	2.475	0.273	6.153

Investigating the distribution of electron densities is a concept that leads to the charge transfer process. Equation (7) gives the charge transfer process, which takes into account the Mullikin charges of the surfaces before and after adsorption.

(7) $$Q_{t}=Q_{\text{adsorption}}-Q_{\text{isolated}}$$

According to the literature, a negative *Q*_t_ value indicates electron transport from gas to the surface, whereas a positive *Q*_t_ value indicates electron transfer from the surface to the gas [62]. According to Table 2, the recorded *Q*_t_ values indicate that electrons are transported from the surfaces under investigation to the molecules of C_2_H_4_, CO, and H_2_S. Positive charge transfer indicates excellent sensor behavior, as described in [63].

### 3.3 Molecular Orbital Studies

The frontier molecular orbitals (FMOs) in a molecule are composed of its highest occupied molecular orbital (HOMO) alongside its lowest unoccupied molecular orbital (LUMO). These orbitals constitute essential tools that enable scientists to determine and anticipate molecules’ physical properties and chemical reactivity [64, 65]. Within molecules, HOMO acts as a site for electron donation, but LUMO serves as the electron acceptance site. Frontier molecular orbitals are fundamental elements of computational chemistry that underpin frontier orbital theory and charge–transfer understanding in chemical reactions. Calculating the energy gap mainly involves considering the energies of the LUMO and HOMO. The energy gap is the difference between the HOMO and LUMO energies. Table 3 shows the calculated energy gap for the systems studied in this research. Figure 2 shows the HOMO–LUMO distribution before adsorption and after adsorption. The computed energy gap values for BC and SiC were 6.545 and 3.090 eV, respectively, prior to adsorption. After adsorption of toxic gases, the energy gap values tend to deviate from their original values due to changes in the electronic structure of the newly formed systems.

FIGURE 2Results of iso‐surfaces of HOMO and LUMO orbitals plotted using VMD software package.

**Figure:**
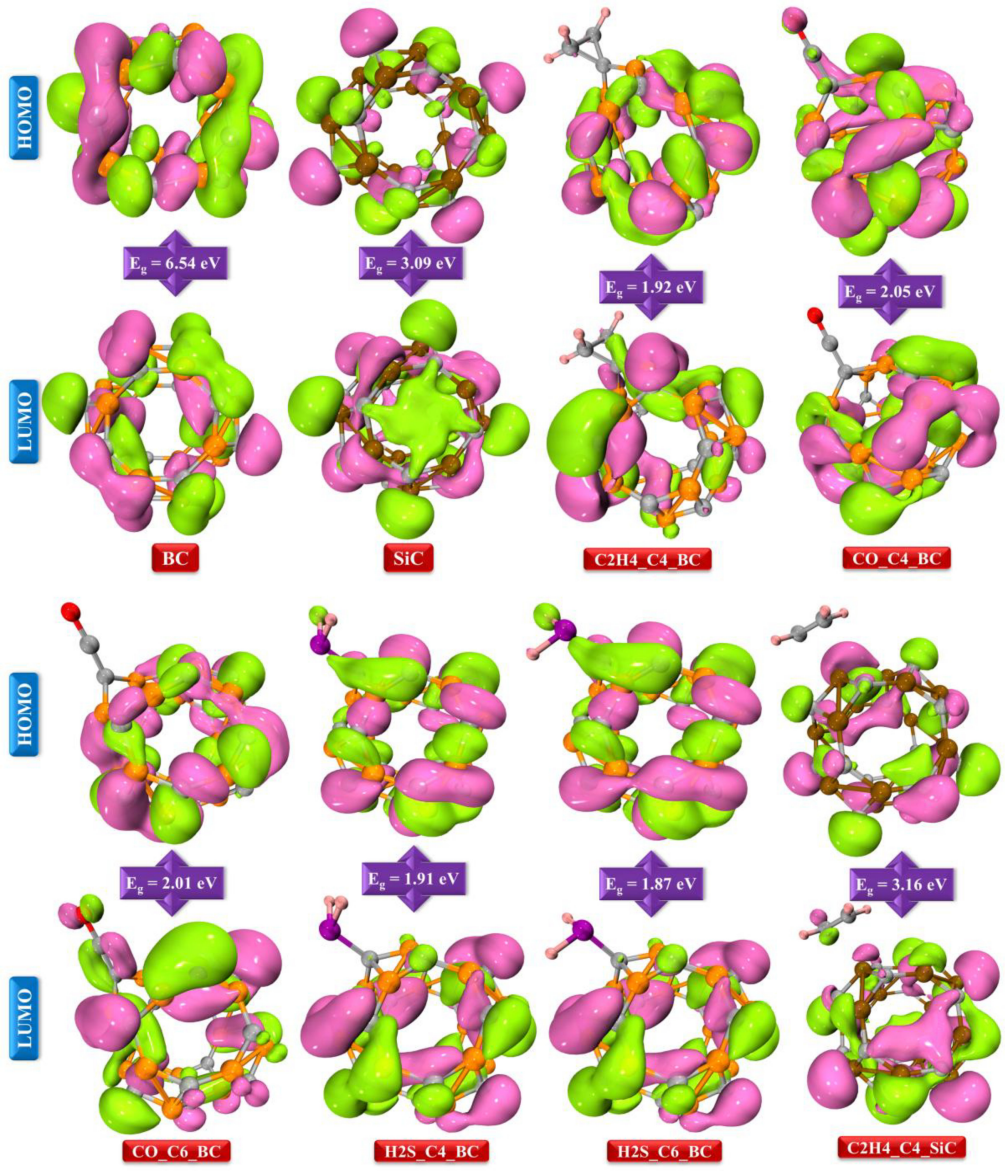

**Figure:**
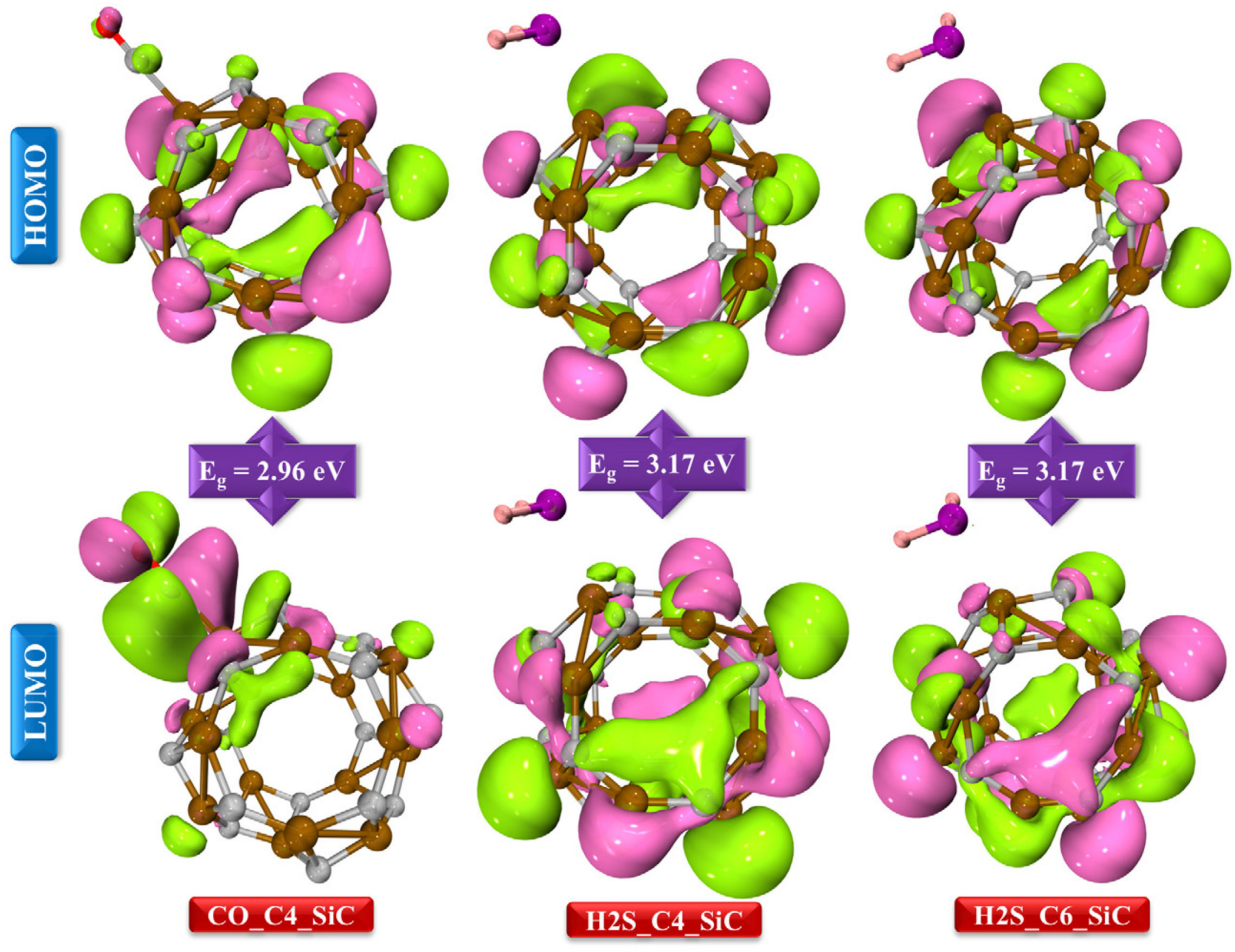

**TABLE 3:** Results of computed value for HOMO, LUMO, energy gap (*E*_g_), the percentage change in energy gap (%Δ*E*_g_), and Fermi energy for all systems.

Systems	HOMO, eV	LUMO, eV	*E*_g_, eV	%Δ*E*_g_	Fermi *E* (*E*_f_), eV
BC	−7.738	−1.194	6.545	—	−4.466
SiC	−6.095	−3.005	3.090	—	−4.550
C_2_H_4__C4_BC	−6.014	−4.092	1.923	−70.618	−5.053
CO_C4_BC	−6.157	−4.111	2.045	−68.747	−5.134
CO_C6_BC	−6.189	−4.181	2.009	−69.308	−5.185
H_2_S_C4_BC	−5.570	−3.656	1.914	−70.755	−4.613
H_2_S_C6_BC	−5.599	−3.732	1.867	−71.478	−4.665
C_2_H_4__C4_SiC	−5.834	−2.675	3.159	2.228	−4.254
CO_C4_SiC	−5.948	−2.988	2.960	−4.183	−4.468
H_2_S_C4_SiC	−5.791	−2.621	3.170	2.589	−4.206
H_2_S_C6_SiC	−5.801	−2.631	3.170	2.589	−4.216

**FIGURE 3:**
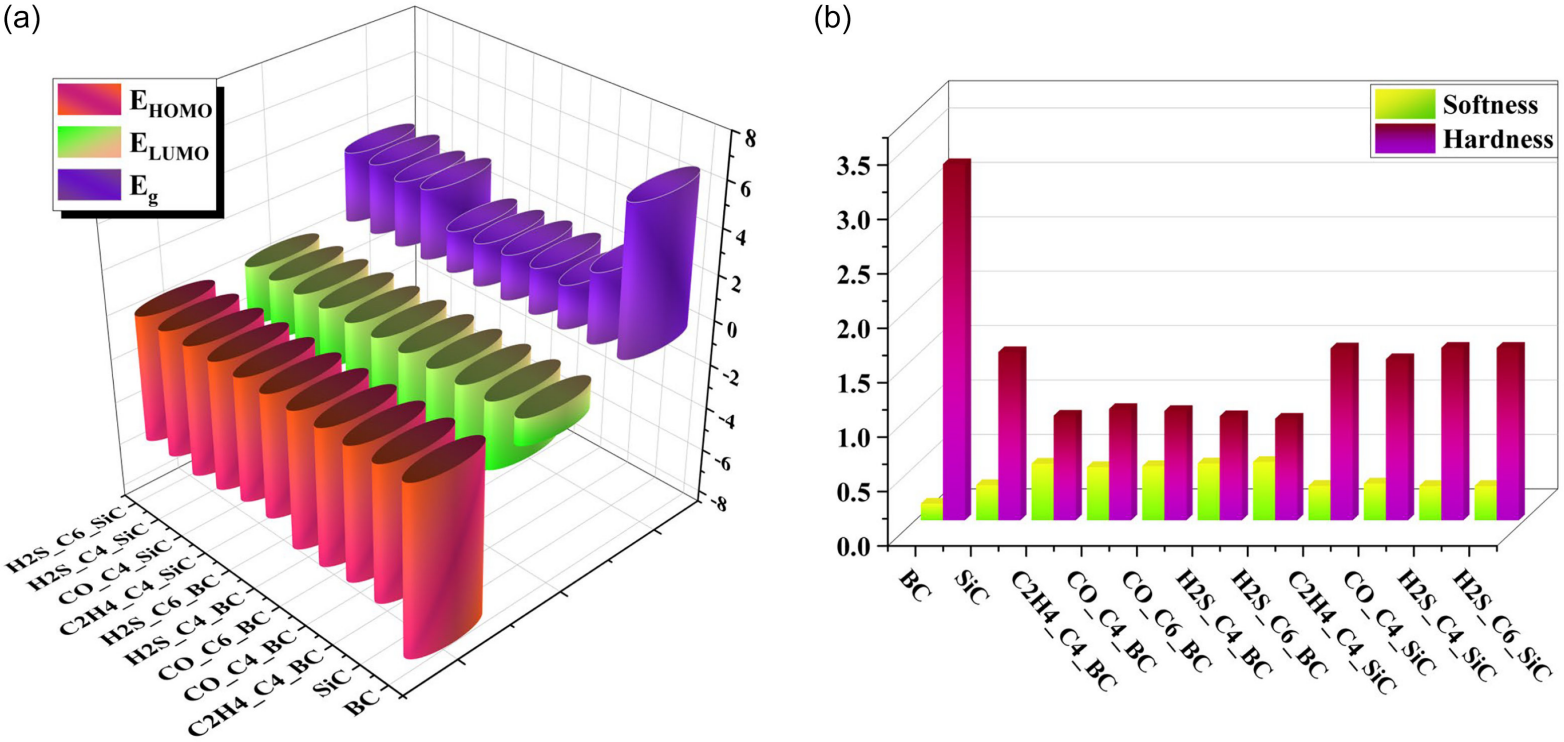
Comparative study of (a) HOMO, LUMO, and energy gap and (b) chemical hardness and chemical softness for all systems.

Among the studied systems, BC systems have the least energy gap values compared with SiC systems. The energy gap values significantly decrease after gases are adsorbed on boron carbide (BC) nanocages. This decrease signifies the high reactivity of the nanocage surface towards toxic gases. The largest decrease in energy gap (1.867 eV) was observed in the H_2_S_C6_BC complex, with a percentage decrease of −71.478% relative to the bare BC nanocage. CO_C4_BC system has the largest energy gap among BC systems, with a value of 2.045 eV and an overall decrease of −68.747% compared with the BC nanocage. The energy gap values for BC systems decrease in the order: CO_C4_BC > CO_C6_BC > C_2_H_4__C4_BC > H_2_S_C4_BC > H_2_S_C6_BC. On the other hand, the energy gap values increase for SiC systems after adsorption, except for one system. The energy gap decrease was calculated for CO_C4_SiC, which had an *E*_g_ of 2.960 eV and a percentage decrease of −4.183% compared with bare SiC. The energy gap for the remaining systems is widening, indicating reduced reactivity of SiC nanocages toward these toxic gases. The comparison of HOMO, LUMO, and energy gap is presented in Figure 3a. Overall, the decrease in energy gap among BC systems shows its high reactivity towards poisonous gases, which is suitable for sensing performance. On the other hand, the lower reactivity of SiC systems is not suitable for sensing applications due to the high energy gap.

### 3.4 Quantum Chemical Descriptors

Quantum chemical descriptors are invaluable measurements that can characterize a system’s stability, sensitivity, conductivity, and reactivity. For computational work, they are a fundamental approach to understanding a system’s optical and electrical properties [66, 67]. The quantum chemical descriptors were calculated using the B3LYP‐D3/6‐31G(d, p) method to better understand the stability and reactivity of the different surfaces. Thanks to the reactivity descriptors, we now know more about the chemical reactivity and kinetic stability of the tagged systems. Chemical quantum descriptors, including ionization potential (IP), electron affinity (EA), chemical softness (*σ*), electrophilicity (*ω*), chemical potential (*μ*), and chemical hardness (*η*), have been the subject of theoretical research to evaluate the reactivity features of the surfaces being studied. The outcomes of these quantum descriptors’ calculations are presented in Table 4. The ionization potential and electron affinity values are calculated in Equations (8) and (9).

**TABLE 4:** Quantum chemical descriptors were calculated for all systems. All values are in eV units.

Systems	IP, eV	EA, eV	*σ*, eV	*η*, eV	*μ*, eV	*ω*, eV
BC	7.738	1.194	0.153	3.272	−4.466	3.048
SiC	6.095	3.005	0.324	1.545	−4.550	6.700
C_2_H_4__C4_BC	6.014	4.092	0.520	0.961	−5.053	13.278
CO_C4_BC	6.157	4.111	0.489	1.023	−5.134	12.887
CO_C6_BC	6.189	4.181	0.498	1.004	−5.185	13.384
H_2_S_C4_BC	5.570	3.656	0.522	0.957	−4.613	11.119
H_2_S_C6_BC	5.599	3.732	0.536	0.933	−4.665	11.661
C_2_H_4__C4_SiC	5.834	2.675	0.317	1.579	−4.254	5.730
CO_C4_SiC	5.948	2.988	0.338	1.480	−4.468	6.743
H_2_S_C4_SiC	5.791	2.621	0.315	1.585	−4.206	5.582
H_2_S_C6_SiC	5.801	2.631	0.315	1.585	−4.216	5.607

(8) $$\mathrm{IP}=-E_{\mathrm{HOMO}}$$

(9) $$\mathrm{EA}=-E_{\mathrm{LUMO}}$$

Ionization potential is the amount of energy needed to transfer an electron from the HOMO orbital to the LUMO orbital of systems. Electron affinity is the amount of energy released from the system after the addition of an electron to it. The electrophilicity index defines the ability of systems to receive electrons. Chemical hardness and chemical softness are opposites: Higher softness values indicate high reactivity, while higher hardness values indicate the system’s stability and resistance to change. The bare BC nanocage has chemical softness and hardness values of 0.153 and 3.272 eV, respectively. After gas adsorption, the chemical softness values increase in BC systems, while the chemical hardness values decrease. The comparison between chemical softness and chemical hardness is given in Figure 3b. The increased chemical softness is related to high reactivity, ultimately aiding gas‐sensing. The most reactive system among BC complexes is H_2_S_C6_BC, with a chemical softness of 0.536 eV and chemical hardness of 0.933 eV. H_2_S_C4_BC is the second most reactive system, with a softness of 0.522 eV and a global hardness of 0.957 eV. On the other hand, only one of the four SiC systems shows greater reactivity than SiC. Only CO_C4_SiC has a higher chemical softness of 0.338 eV and a lower chemical hardness of 1.480 eV. The remaining three systems are less reactive toward the toxic gases under study. This is mainly due to the increase in the energy gap after the adsorption of analytes on SiC nanocage. The BC complexes exhibit greater reactivity and responsiveness to gases than SiC complexes. Moreover, the electrophilicity values for BC systems are also higher than those for SiC systems. The highest electrophilicity is observed in CO_C6_BC, with a value of 13.384 eV. BC systems also have lower chemical potential values than SiC systems, which helps increase their reactivity.

### 3.5 Density of State (DOS) Studies

The number of possible states at each energy level of a system can be determined using the density of states (DOS) analysis. DOS enables the gathering of data on the conductivity of the surfaces being studied [68]. The number of occupied states is one direct correlation between the system’s dispersion characteristics and a high state density at a particular level [69]. Figure 4 shows the surface and interface DOS graphs before and after adsorption of C_2_H_4_, H_2_S, and CO. For each DOS plot, the upper border axis shows the total density of states (TDOS), and the other line in the spectrum shows the partial density of states (PDOS) and the interactions among the different fragments. The various Fermi energy (*E*_f_) ranges deduced from the graphs illustrate the unique features of the electronic density for each orbital in the systems being studied. The Fermi energy levels of BC and SiC before adsorption are −4.47 and −4.55 eV, respectively, as shown in Table 3. Adsorption resulted in significant changes in electronic properties, and BC was more sensitive to adsorption than SiC. For the BC systems, CO adsorption showed the most substantial decrease in Fermi energy, −5.134 eV for CO_C4_BC and −5.185 eV for CO_C6_BC, thereby verifying the strong electron‐withdrawing character of CO and the improved sensing performance. In contrast, the Fermi energy was moderately reduced upon H_2_S and C_2_H_4_ adsorption on BC, implying a relatively weaker electronic interaction than that of CO.

FIGURE 4The density of states (DOS) spectra for bare BC and SiC as well as all the designed systems under study.

**Figure:**
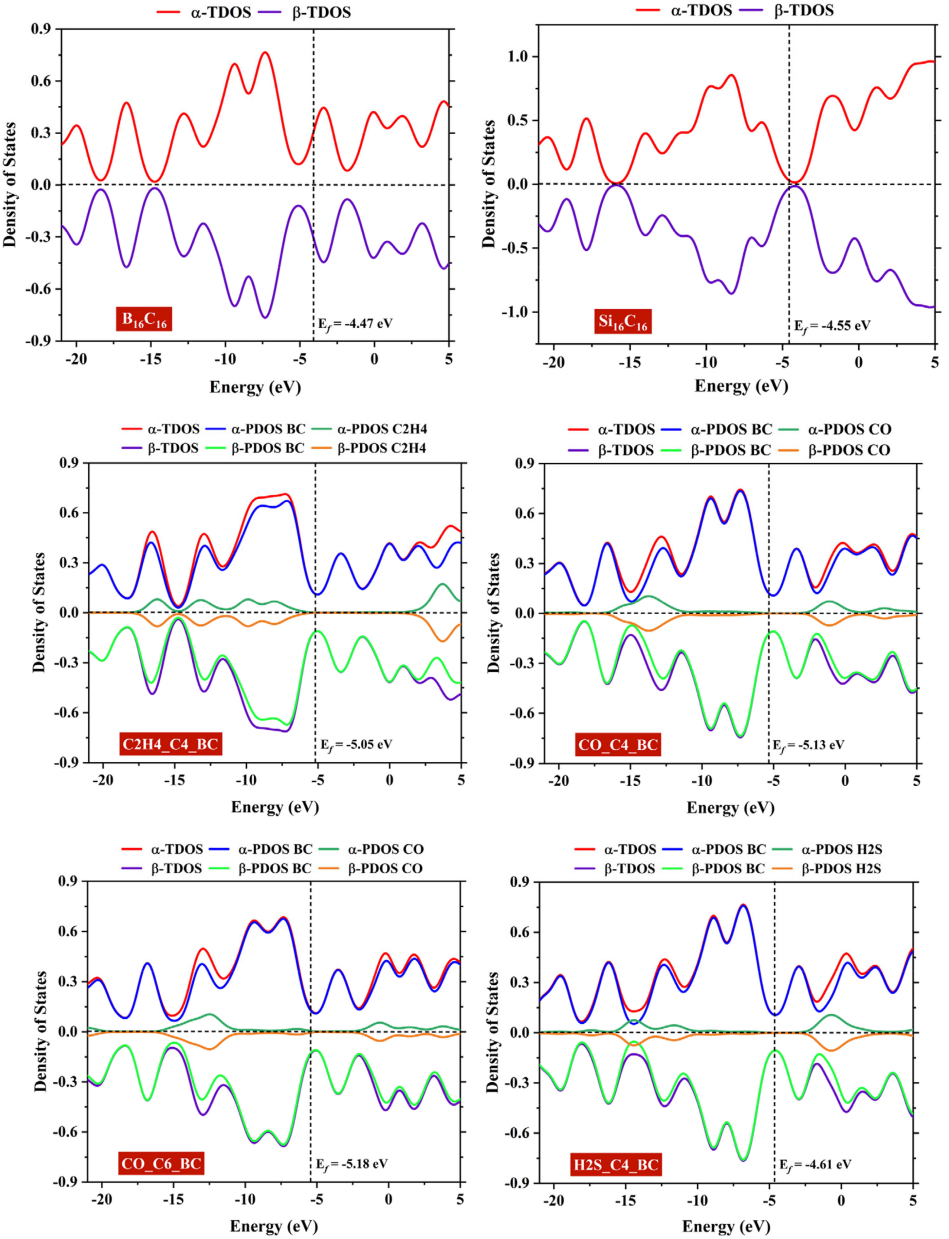

**Figure:**
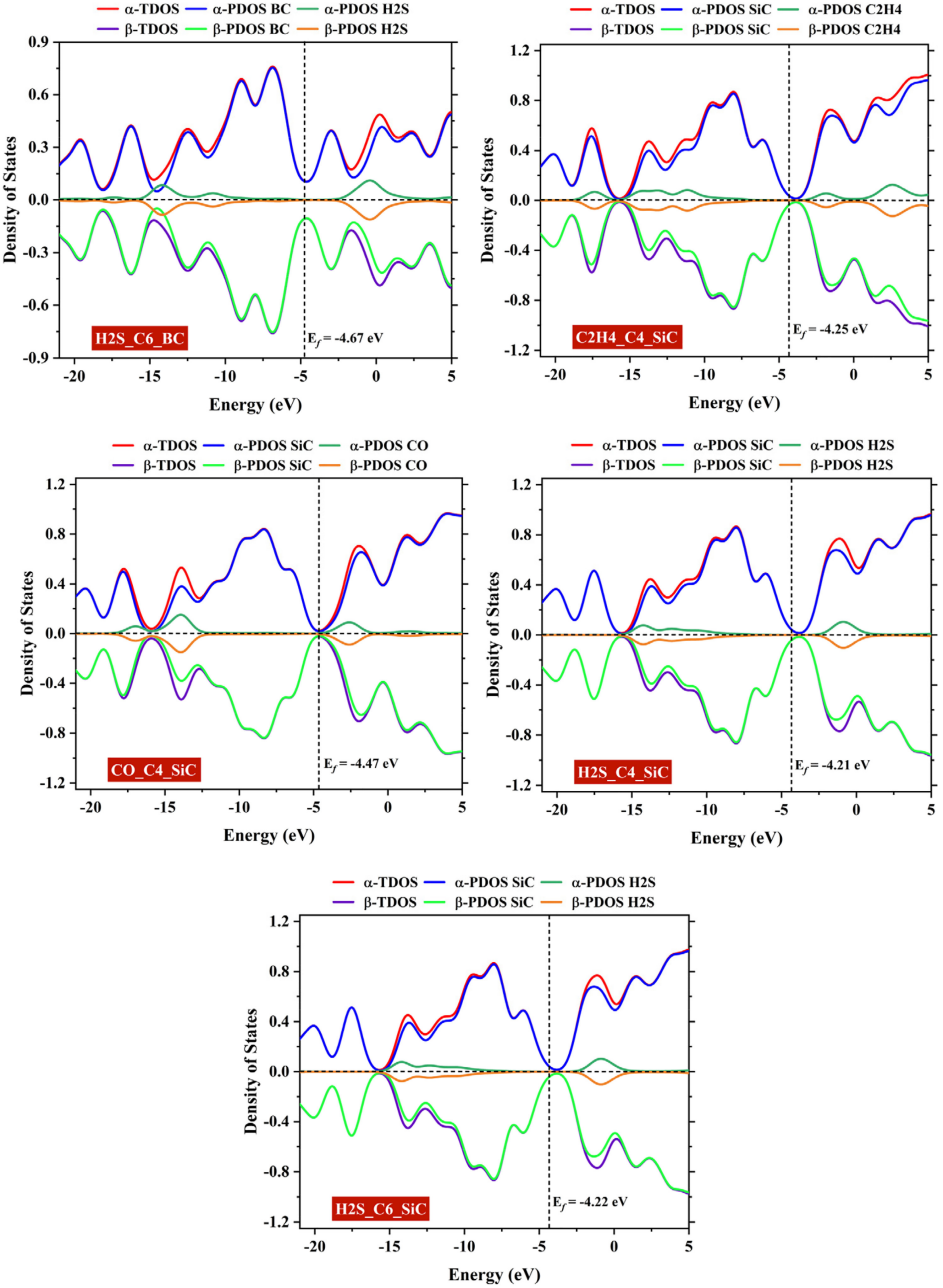

For SiC, the magnitude of the Fermi energy change brought by adsorption was significantly smaller, and its Fermi energy is less sensitive to the effects of electron withdrawal or donating moieties. In contrast, the Fermi energy of CO_C4_SiC could reach −4.468 eV adsorbed with CO, whereas adsorption with either H_2_S or C_2_H_4_ had minimal effect, and their Fermi energy came closer to that of its parent molecule. These findings indicate that SiC might exhibit a significantly lower tendency to change its electronic properties upon adsorption. The outcomes indicate that for BC systems, the Fermi energy can be tailored with high precision utilizing selective adsorption, and CO at C6 provided the minimum Fermi energy (−5.185 eV).

### 3.6 NBO Studies

NBO analysis is recognized as a key approach for providing information on the electron distribution within molecular bonds between diverse atomic species. The second‐order perturbation energy has been used to learn about intramolecular and intermolecular charge transport [70]. Table 5 shows the findings utilizing the second‐order perturbation energy for the natural bonding orbital (NBO). The charge transfer pattern in a surface may likewise be shown using this study [71]. In addition, NBO analysis provides the valence space information between the virtual and occupied Lewis’s orbitals and the kind of orbital, interactions, and occupancy level [72]. The stabilization or perturbation energy may be determined from the relationship shown in Equation (10).

**TABLE 5:** Studies of NBOs show interactions between donor and acceptor orbitals and the associated stabilization energies.

Systems	Donor	Acceptor	*E* ^2^, kcal mol^−1^	*E*(*j*)–*E*(*i*) a.u.	*F*(*j*,*i*) a.u.
C_2_H_4__C4_BC	*σ* B_20_‐C_21_	σ* C_21_‐B_22_	9.96	0.94	0.09
	*π* C_5_‐B_6_	σ* B_4_‐C_5_	7.51	0.58	0.13
	LP C_7_	π* C_5_‐B_6_	9.28	0.23	0.086
	LP C_17_	σ* C_17_‐B_27_	4.23	0.51	0.069
CO_C4_BC	*σ* C_3_‐B_13_	σ* B_4_‐C_5_	9.77	0.93	0.086
	*π* O_33_‐C_34_	σ* C_21_‐B_25_	0.8	0.75	0.022
	LP O_33_	σ* C_21_‐C_34_	9.56	1.37	0.102
	LP C_26_	σ* B_15_‐C_26_	7.31	0.57	0.081
CO_C6_BC	*σ* B_25_‐C_26_	π* O_33_‐C_34_	11.37	0.53	0.07
	*σ* C_1_‐B_11_	σ* B_2_‐C_3_	10.45	0.94	0.089
	*π* C_26_‐C_34_	π* C_26_‐C_34_	7.46	0.32	0.045
	LP O_33_	π* C_26_‐C_34_	100.85	0.34	0.168
H_2_S_C4_BC	*σ* C_7_‐B_15_	σ* C_1_‐B_8_	15.49	0.94	0.108
	*σ* C_3_‐B_13_	σ* B_4_‐C_5_	10.66	0.93	0.089
	LP C_1_	σ* C_1_‐B_11_	4.42	0.52	0.069
H_2_S_C6_BC	*σ* C_3_‐B_13_	σ* B_4_‐C_5_	17.09	0.94	0.114
	*σ* C_7_‐B_15_	σ* C_1_‐B_8_	10.66	0.93	0.089
	LP C_3_	σ* B_2_‐C_3_	4.06	0.58	0.062
C_2_H_4__C4_SiC	*σ* C_19_‐Si_27_	σ* Si_20_‐C_21_	11.16	0.77	0.083
	*σ* C_33_‐C_36_	π* Si_13_‐C_14_	6.00	0.8	0.067
	*π* C_7_‐Si_9_	π* C_1_‐Si_8_	36.62	0.25	0.087
CO_C4_SiC	*σ* C_17_‐Si_31_	π* C_16_‐Si_31_	11.79	0.55	0.077
	*π* Si_27_‐C_28_	π* C_5_‐Si_13_	36.62	0.26	0.088
	LP C_34_	π* C_16_‐Si_31_	116.37	0.5	0.217
	LP C_34_	σ* C_17_‐Si_31_	33.90	0.67	0.144
H_2_S_C4_SiC	*σ* C_3_‐Si_11_	σ* Si_4_‐C_5_	11.53	0.78	0.085
	*π* C_1_‐Si_2_	π* Si_15_‐C_26_	41.25	0.26	0.094
	LP S_33_	π* C_3_‐Si_11_	73.51	0.44	0.164
	LP S_33_	σ* Si_11_‐C_32_	10.25	0.65	0.077
H_2_S_C6_SiC	*σ* C_19_‐Si_27_	σ* Si_20_‐C_21_	11.29	0.79	0.084
	*π* Si_29_‐C_30_	π* Si_22_‐C_23_	32.44	0.25	0.083
	LP S_33_	π* Si_27_‐C_28_	74.23	0.44	0.164

(10) $$E^{2}=\Delta E_{i,j}-q\frac{F^{2}(i,j)}{E(\text{−}E)}$$

According to Equation (10), the Donor occupancy is denoted by *q*, the diagonal elements are *E* *_i_* and *E* *_j_*, and the Fock matrix element is *F  *(*i*,  *j*). Table 5 shows the summarized second‐order perturbation energies *E* ^2^ of the donor–acceptor interacting NBOs in the considered complexes. Several studies have shown that more considerable stabilization energy indicates more conjugation and a high level of contact between the donor and acceptor orbitals [73]. Among the studied systems, the largest stabilization energy is calculated for CO_C4_SiC, having a value of 116.37 kcal/mol. This high stabilization energy results from the transition of lone pair electrons of C_34_ to the anti‐bonding *π* orbital of the C_16_─Si_31_ bond. H_2_S_C6_SiC has a stabilization energy of 74.23 kcal/mol, which is also due to the lone pair transition from a sulfur atom of H_2_S to the anti‐bonding *π* orbital of the Si_31_─C_28_ bond of SiC nanocage. Another higher stabilization energy of 73.51 kcal/mol is present in H_2_S_C4_SiC due to the movement of the lone pair electron of S_33_ to the anti‐bonding *π* orbital of the C_3_─Si_11_ bond. The stabilization energies of BC systems are relatively lower than those of SiC systems, as seen in Table 5. The highest stabilization energy is observed in one system with CO adsorption on BC nanocage. CO_C6_BC has the computed stabilization energy value of 100.85 kcal/mol due to the transition of the lone pair electron of O_33_ of the CO gas to the anti‐bonding *π* orbital of the C_26_─C_34_ bond of the BC nanocage. H_2_S_C6_BC has the second highest stabilization energy among BC systems after the adsorption of H_2_S gas on BC nanocage. It has 17.09 kcal/mol of the stabilization energy due to electron transition from the bonding *σ* orbital of the C_3_─B_13_ bond to the anti‐bonding *σ* of the B_4_─C_5_ bond. The overall positive and high stabilization energies highlight the higher sensing potential arising from strong interactions between the donor and acceptor orbitals across all designed systems.

### 3.7 QTAIM Studies

**FIGURE 5:**
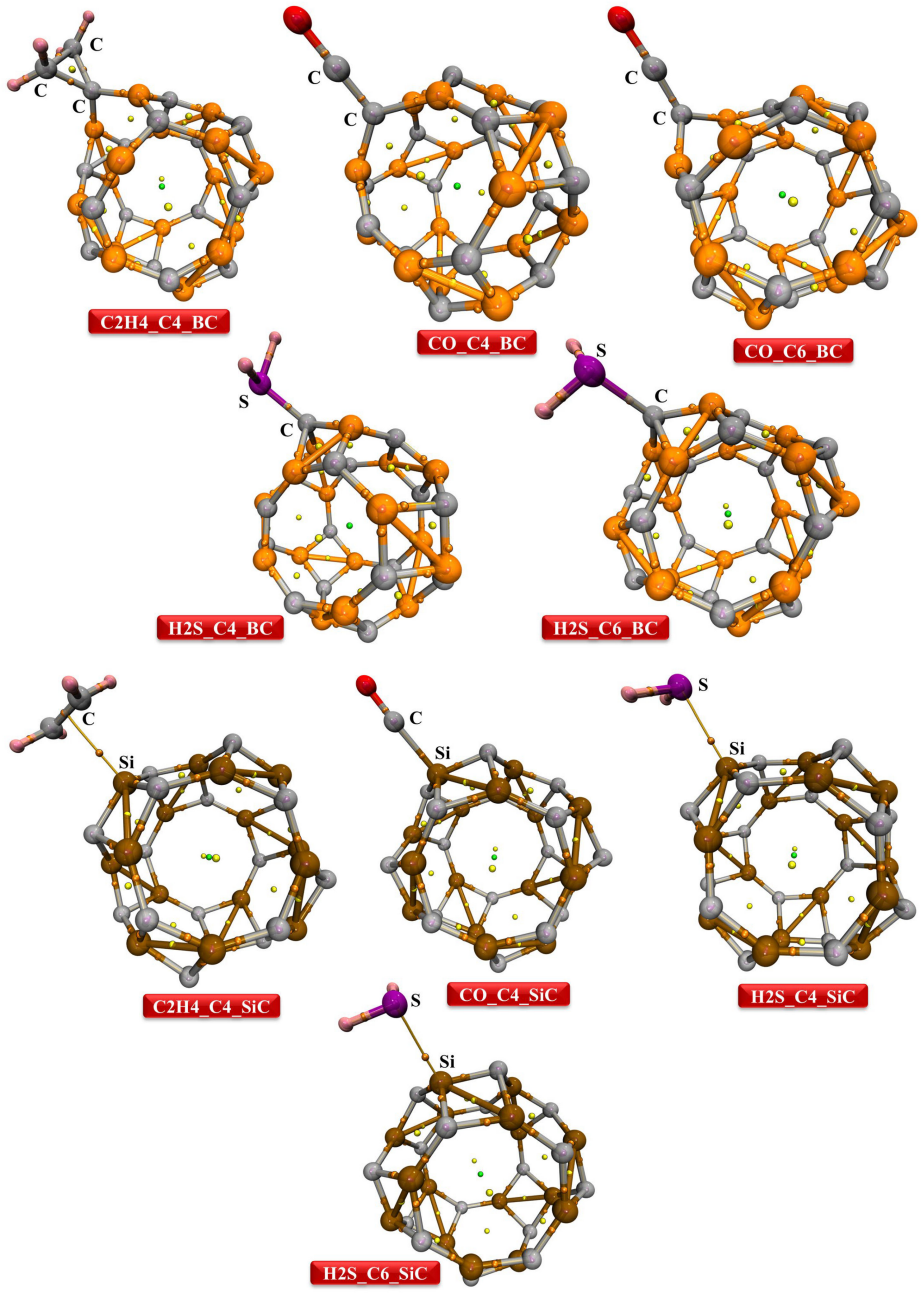
The 3D iso‐surface plots of QTAIM are used to study the interaction between the adsorbate and the adsorbent.

Obtaining information about the types of bonding between atoms, details about inter‐ and intramolecular connections, and the electron density distribution of surfaces and adsorbents is crucial for characterizing the binding capacity of a surface with the adsorbate and estimating the unique interactions between the systems [63, 74]. So, assessing how strong the hydrogen bonding will be between the surfaces is essential. In this study, atoms‐in‐molecules (AIM) analysis enables a comprehensive topological analysis to determine the electron density values and bond properties [75]. To study the structures, molecular properties, nature of interactions, and electron distributions of the system, Bader et al. postulated the AIM analysis, which is the most used theory for investigating hydrogen bonds at the structural unit [76]. According to the theory, the series of interactions between two atoms is called the bond path (BP), and the place on the BP with the densest concentration of electrons is called the bond critical point (BCP). The different parameters of topology, including the Laplacian of electron density (∇^2^ *ρ*(*r*)), kinetic energy density (*G*(*r*)), potential energy density (*V*(*r*)), total electron energy density (*H*(*r*)), and electron density ( *ρ*(*r*)) are reviewed in this study using the multifunctional wave analyzer (Multiwfn). The QTAIM outcomes of different systems used in this study are highlighted in Table 6. The electron density ( *ρ*(*r*)) values predict the bond formation strength and possibility between adsorbate and adsorbent. All electron density values are positive, suggesting the strong bond formation and interaction between the two surfaces. The nature of the bond formed is predicted using the values of Laplacian of electron density (∇^2^ *ρ*(*r*)) and total electron energy density (*H*(*r*)). The noncovalent nature of interactions is presented with ∇^2^ *ρ*(*r*) positive or more than zero and *H*(*r*) positive or more than zero. The covalent nature of the bond has both these values, which are less than zero or negative. The bond will be partially covalent if ∇^2^ *ρ*(*r*) values are positive while *H*(*r*) is negative. In this study, most of the systems have ∇^2^ *ρ*(*r*) values, which are negative, while some positive values of ∇^2^ *ρ*(*r*) are also present. On the other hand, all the systems have negative values of *H*(*r*). Based on this observation, the bond forming in our systems is primarily covalent, while some partial covalent bonds also participate in the interaction process. The values of the electron localized function (ELF) predict the delocalization of electrons in the studied system. ELF values of 0 represent the maximum electron delocalization, while maximum electron localization has ELF values of 1 [77]. In our designed systems, almost all the values of ELF are under 1, suggesting the considerable delocalization of electrons. SiC systems have the highest electron delocalization due to their values under 0.5 compared to BC systems. Bond ellipticity (*ε*) values are used for the stability and instability of bonds formed during the interaction process. A value of *ε* under 1 represents bond stability, while more than 1 represents bond instability. Most of our designed systems have ellipticity values of less than 1, while only one system has a value of more than 1 which suggests that most of the bonds formed in our systems are stable. Overall, the designed systems have a covalent or partial covalent nature of interactions, and electron delocalization occurs. The 3D iso‐surface representation of the QTAIM study is revealed in Figure 5.

**TABLE 6:** Various topological parameters were calculated at bond critical points (BCPs) using the DFT B3LYP‐D3 method. All electron density and energy density values (*ρ*(*r*), ∇²*ρ*(*r*), *G*(*r*), *V*(*r*), and *H*(*r*)) are given in atomic units (a.u.). *V*(*r*)/*G*(*r*), ELF, and bond ellipticity (*ε*) are dimensionless quantities.

Systems	Connections	BCP	*ρ*(*r*) (a.u.)	∇^2^ *ρ*(*r*) (a.u.)	*G*(*r*) (a.u.)	*V*(*r*) (a.u.)	*H*(*r*) (a.u.)	*V*(*r*)/*G*(*r*)	ELF	*ε*
C_2_H_4__C4_BC	C_10_‐C_36_	60	0.234	−0.401	0.086	−0.273	−0.186	3.161	0.897	0.415
	C_10_‐C_33_	75	0.223	−0.341	0.087	−0.259	−0.172	2.985	0.881	0.514
CO_C4_BC	C_21_‐C_43_	74	0.315	−0.643	0.327	−0.815	−0.488	2.491	0.621	0.139
CO_C6_BC	C_26_‐C_34_	66	0.321	−0.662	0.334	−0.834	−0.500	2.495	0.626	0.246
H_2_S_C4_BC	C_16_‐S_33_	64	0.194	−0.291	0.072	−0.217	−0.145	3.010	0.871	0.088
H_2_S_C6_BC	C_14_‐S_33_	62	0.199	−0.310	0.074	−0.225	−0.151	3.049	0.875	0.103
C_2_H_4__C4_SiC	Si_13_‐C_33_	73	0.036	0.017	0.011	−0.018	−0.007	1.628	0.505	1.715
CO_C4_SiC	Si_31_‐C_34_	65	0.068	0.198	0.075	−0.101	−0.026	1.342	0.156	0.027
H_2_S_C4_SiC	Si_11_‐S_33_	78	0.051	−0.001	0.023	−0.046	−0.023	2.013	0.444	0.087
H_2_S_C6_SiC	Si_27_‐S_33_	65	0.051	−0.003	0.022	−0.044	−0.023	2.032	0.454	0.080

### 3.8 Reduced Density Gradient (RDG) Studies

**FIGURE 6:**
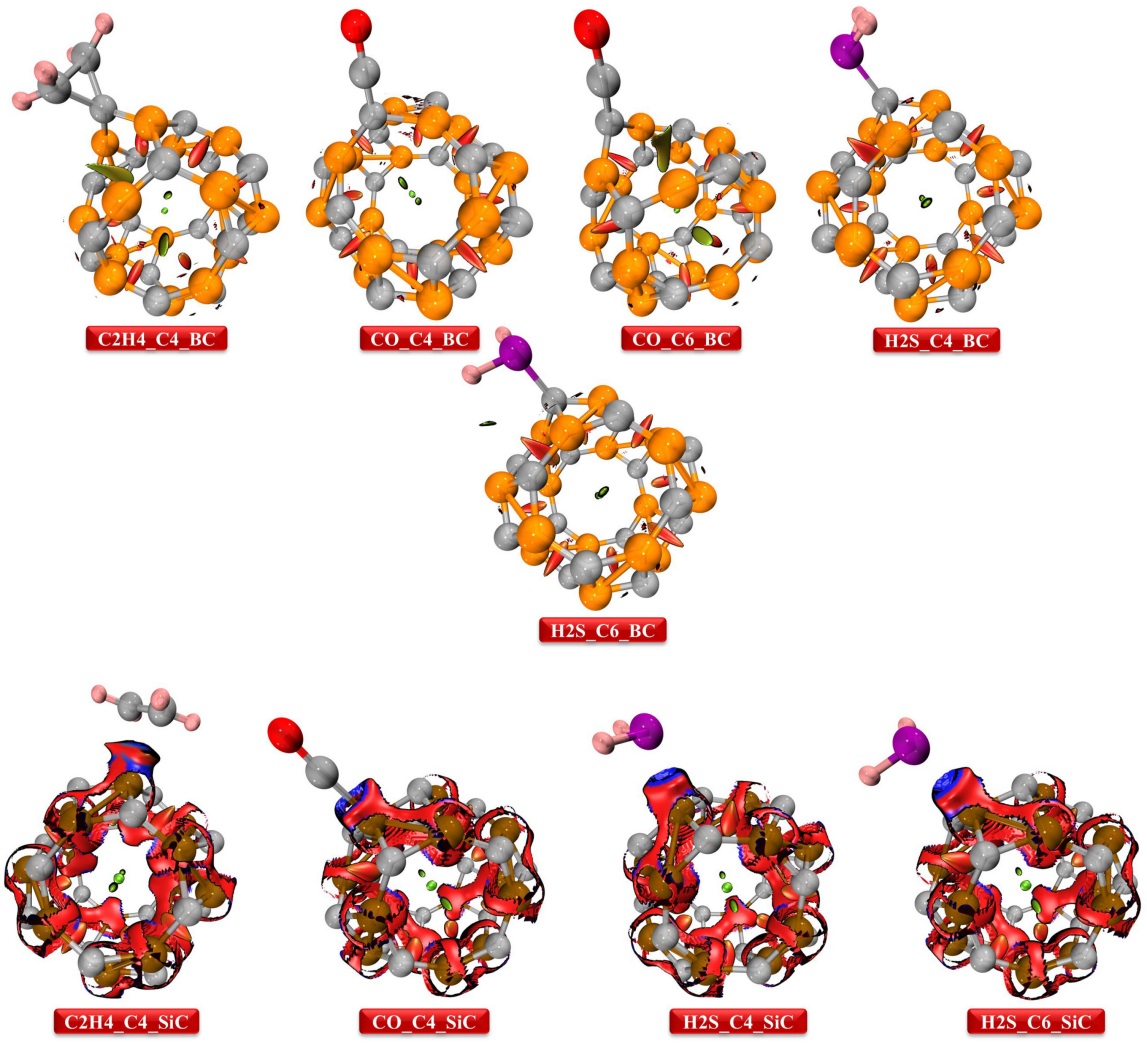
3D iso‐surfaces for the adsorption of C_2_H_4_, CO, and H_2_S on SiC and BC nanocages.

The study of noncovalent interactions is essential for several reasons, including their influence on the chemical and physical behavior of different surfactants [78]. The attractive interactions between electronegative proton donors and acceptors in hydrogen bonding are a key component of this interaction [79]. Electrostatic forces, such as van der Waals interactions, also play a role in the noncovalent interaction (NCI) analysis. The noncovalent interaction (NCI) research uses the reduced density gradient (RDG) as a parameter to analyze weak interactions in real space. This parameter makes it possible for quick and easy comprehension of a system’s noncovalent properties. The RDG plots of this investigation and the analysis’s outcome are shown by multiplying the second eigenvalue of the Hessian matrix (*λ*2). Figure 6 shows 3D iso‐surface plots and Figure S1 represents the 2D‐RDG graphs plotted using the Multiwfn and VMD software. To analyze the interaction between the nanocages and the gases, color changes in the 2D‐RDG scatter plots are used. On the other hand, the nature of the interactions within the complexes is shown by the 3D isosurfaces. The negative sign of the second eigenfunction explicitly indicates strong attraction, as shown in blue. The green zone represents the Van der Waals force, a weak interaction that becomes noticeable when the second eigenvalue is near zero. The 2D‐RDG graphs show that the complexes have a higher mass of red color, suggesting a greater presence of steric repulsion forces. All of our designed systems have prominent blue regions, which signify the presence of strong, attractive forces, as predicted by the previous QTAIM study. The presence of relatively few patches introduces the Van der Waals forces in designed systems. The 3D iso‐surfaces of SiC complexes demonstrate strong repulsive forces compared to BC complexes.

### 3.9 Sensor Mechanism

Sensor mechanisms can be used to measure the performance of studied nanocages for the C_2_H_4_, CO, and H_2_S toxic gases. It includes different parameters like electrical conductivity (*σ*), work function (Φ), recovery time (*τ*), and sensing response (*S*).

#### 3.9.1 Electrical Conductivity (*σ*)

The electrical conductivity (*σ*) is a measure of the electron flow from HOMO to the LUMO orbitals of systems, and it depends upon the energy gap of that system. The sensing performance of a system depends upon the changes in its electrical conductivity [80, 81, 82]. At room temperature, the electrical conductivity can be given by Equation (11).

(11) $$\text{σ}={\mathrm{AT}}^{3/2}\exp\;\left(\frac{-E_{g}}{2\mathrm{KT}}\right)$$

**FIGURE 7:**
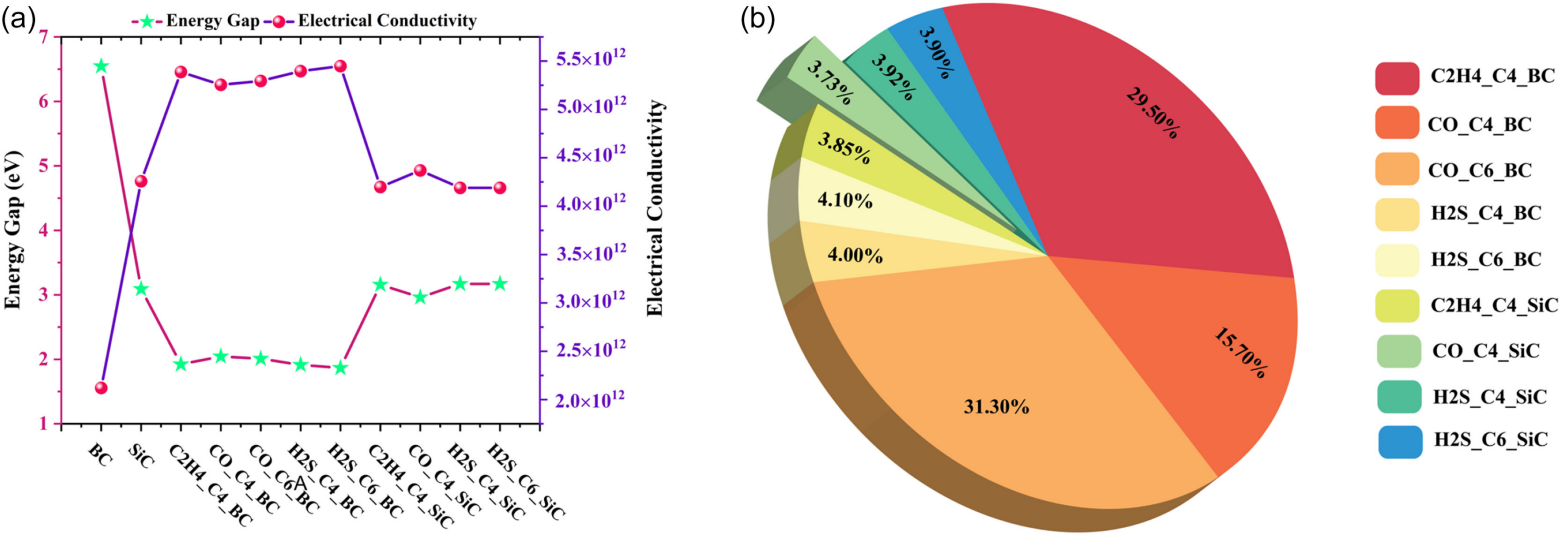
Graphical representation of (a) comparison between energy gap and electrical conductivity and (b) percentage recovery time for each of the designed systems.

Herein, A is called the Richardson constant (6 × 10^5^ A m^−^^2^ K^−2^), T is the working temperature (298 K) of the systems, and *K* is called the Boltzmann constant (8.318 × 10^−^^3^ kJ mol^−^^1^ K^−1^). Using this equation, the calculated electrical conductivity values are listed in Table 7. The studied systems display measurable changes in electrical conductivity (*σ*) values following functionalization because the chemical modifications alter the charge transport properties. The basic materials BC and SiC both display electrical conductivities at 2.12 × 10^12^ S/m and 4.26 × 10^12^ S/m, which serve as a reference point to evaluate how functional groups affect the samples. The application of toxic gases to BC structures enhances electrical conductivity in all investigated examples. The sample of H_2_S_C6_BC shows the maximum conductance level at 5.45 × 10^12^ S/m, while H_2_S_C4_BC (5.40 × 10^12^ S/m) and C_2_H_4__C4_BC (5.39 × 10^12^ S/m) come in close second. The results show that H_2_S and C_2_H_4_ modification of BC enhances charge transport by introducing electronic states that contribute to conductance. Building from CO adsorption BC systems yields conductivities of 5.29 × 10^12^ S/m for CO_C6_BC and 5.26 × 10^12^ S/m for CO_C4_BC, although these values fall below those of H_2_S and C_2_H_4_ functionalities. The base SiC system demonstrates a conductivity of 4.26 × 10^12^ S/m because of its higher intrinsic conductivity. Only minimal changes in conductivity occur when C_2_H_4_, CO, and H_2_S functionalize the material. The highest conductivity level of 4.37 × 10^12^ S/m is found in CO_C4_SiC among all functionalized SiC‐based systems because CO performs better at promoting charge transport in SiC than in BC. Two samples of H_2_S_C6_SiC (4.19 × 10^12^ S/m) and H_2_S_C4_SiC (4.19 × 10^12^ S/m) present the lowest conductivity among the SiC‐based compounds due to H_2_S not contributing to significant conductivity improvement in SiC materials. The variations of electrical conductivity and energy gap are given in Figure 7a.

**TABLE 7:** The computed results of the sensing mechanism for all systems, including electrical conductivity (*σ*), work function (Φ), recovery time (*τ*), and sensing response (*S*) (dimensionless).

Systems	*σ*, S/m	Φ, eV	*τ*, sec	*S*
BC	2.12 × 10^12^	4.466	—	—
SiC	4.26 × 10^12^	4.550	—	—
C_2_H_4__C4_BC	5.39 × 10^12^	5.053	116.01	1.540
CO_C4_BC	5.26 × 10^12^	5.134	0.006	1.478
CO_C6_BC	5.29 × 10^12^	5.185	659.03	1.497
H_2_S_C4_BC	5.4 × 10^12^	4.613	1.12 × 10^−8^	1.545
H_2_S_C6_BC	5.45 × 10^12^	4.665	2.22 × 10^−8^	1.569
C_2_H_4__C4_SiC	4.2 × 10^12^	4.254	4.66 × 10^−11^	−0.014
CO_C4_SiC	4.37 × 10^12^	4.468	3.71 × 10^−11^	0.026
H_2_S_C4_SiC	4.19 × 10^12^	4.206	2.01 × 10^−10^	−0.016
H_2_S_C6_SiC	4.19 × 10^12^	4.216	1.68 × 10^−10^	−0.016

#### 3.9.2 Recovery Time (*τ*)

The reusability of a sensor device depends on the sensor’s recovery time. It is an important parameter to consider when designing a sensor, as it determines the time required for a sensor to recover its original shape. An ideal sensor should have a very small recovery time to increase its efficacy and use [83]. The recovery time of a sensor depends on the adsorption energy, which can be calculated using Equation (12).

(12) $$\tau =V_{o}^{-1}\exp\;\left(\frac{-E_{\mathrm{ad}}}{\mathrm{KT}}\right)$$

In this equation, *V*_o_ is the attempt frequency with the value of 10^12^ s^−1^. The observed values demonstrate significant variations between systems because different gas interaction behaviors occur on BC and SiC surfaces. The recovery time values are given in Table 7, and the percentage recovery time for each system is shown in Figure 7b. The CO_C6_BC system requires the longest recovery period of 659.03 s because CO molecules strongly bind to the BC surface. The analytical system of C_2_H_4__C4_BC has a recovery time of 116.01 s, indicating that the stable bond between the surface and the analyte takes longer to break. Alternative sensing materials with H_2_S‐adsorbed BC structures (H_2_S_C4_BC, H_2_S_C6_BC) exhibit instantaneous recovery on nanosecond timescales (1.12 × 10^−^^8^ s and 2.22 × 10^−^^8^ s) due to limited surface binding, which facilitates a prompt response. All functionalized SiC systems operate with ultra‐fast desorption behaviors, resulting in recovery times between 10^−11^ to 10^−^^10^ s because the surface shows minimal binding affinities with adsorbed molecules. C_2_H_4__C4_SiC and CO_C4_SiC function as the fastest sensors due to their rapid rate of recovery at 4.66 × 10^−^^11^ s and 3.71 × 10^−^^11^ s, respectively. These computed results validate BC materials as robust candidates for applications that demand strong adsorption alongside rapid‐release functions (CO_C6_BC along with H_2_S_C4_BC). SiC‐based materials demonstrate naturally quick analyte detection and removal capabilities, which make them superior sensors for monitoring rapid processes.

#### 3.9.3 Work Function (Φ)

The work function (Φ) is the minimum energy required to remove surface electrons into the vacuum. The measurement of work function plays an essential role because it regulates key electronic features and surface interactions, as well as sensor and electronic device usage [84]. The amount of electron binding in a material can be determined by its work function. Strong electron binding leads to less electron emission, but weak electron binding makes it easier for electrons to leave. The work function of a system can be calculated using Equation (13).

(13) $$\Phi =V_{\infty }-E_{f}$$

**FIGURE 8:**
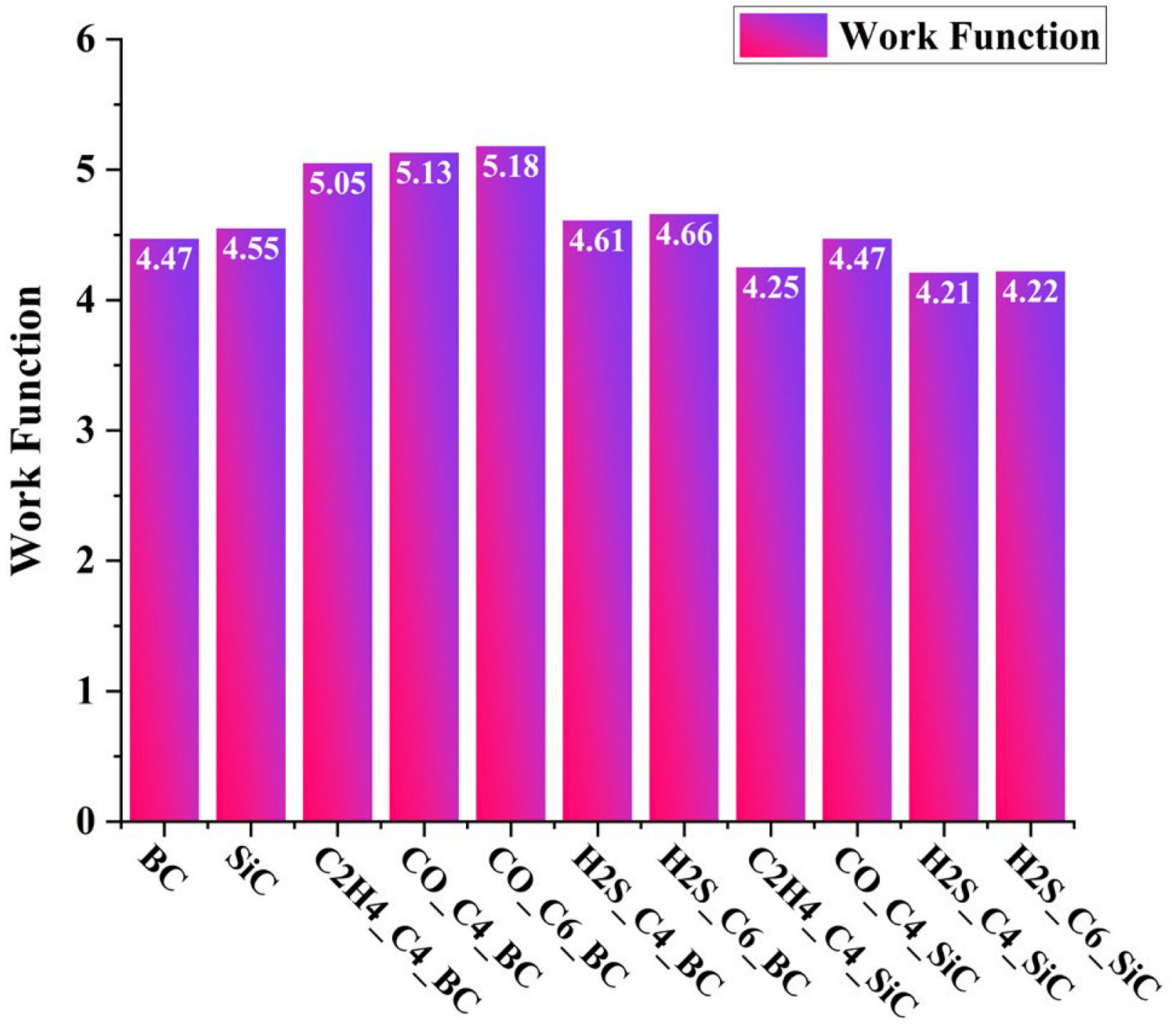
The variations of the work function of all complexes with pristine BC and SiC nanocages.

Herein, *V*_∞_ is the electrostatic potential at the vacuum stage, having an approximate value of zero. The functionalization process raises the work function after CO and C_2_H_4_ adsorption, but provides a more moderate increase in work function with H_2_S adsorption than untreated BC, as shown in Table 7. The work function reaches its maximum values at C_2_H_4__C4_BC (5.053 eV), CO_C4_BC (5.134 eV), and CO_C6_BC (5.185 eV) among all BC‐based systems. A surface electron‐withdrawing effect arises when CO or C_2_H_4_ bonds to the surface, thereby strengthening electron binding and reducing the likelihood of electron emission. SiC‐based materials exhibit different behavior than BC‐based materials, since functionalization consistently lowers their work function. H_2_S‐functionalized SiC systems achieve the lowest work function values among all SiC‐based systems, when they have 4.206 eV for H_2_S_C4_SiC and 4.216 eV for H_2_S_C6_SiC. The variations of work function with BC and SiC nanocages are given in Figure 8. The availability of electrons increases after H_2_S adsorption, because of which material conductivity and surface reactivity improve. The electron transfer properties of SiC improve following H_2_S and C_2_H_4_ functionalization because these molecules decrease the work function.

#### 3.9.4 Sensing Response

The studied functionalized BC and SiC systems exhibit different response patterns in target analyte detection, as reflected in their sensitivities. Sensing performance increases when values are positive, but decreases when values become negative. The sensing response can be calculated based on electrical conductivity using Equation (14)

(14) $$S=\frac{\sigma_{\text{complex}}}{\sigma_{\text{nanocage}}}-1$$

Herein, *σ*_complex_ and *σ*_nanocage_ are the electrical conductivities of the designed complexes and nanocages, respectively. All functionalized BC systems exhibit positive sensing responses, confirming effective detection of the analyzed substances, as shown in Table 7. The highest sensing sensitivity recorded for H_2_S_C6_BC amounts to 1.569, which slightly exceeds the response of H_2_S_C4_BC at 1.545. The data indicate that H_2_S functionalization enables BC to detect targets more effectively due to beneficial electronic interactions and efficient charge–transfer mechanisms. The SiC‐based systems primarily exhibit negative sensing outcomes, indicating a weak response to toxic gases. These H_2_S adsorbed SiC nanomaterials exhibit negative sensing responses of −0.016 (both H_2_S_C4_SiC and H_2_S_C6_SiC), which indicates that these gases fail to enhance SiC detection capabilities or may impede effective sensing outcomes. Among all SiC‐based systems, only CO_C4_SiC shows a minimal positive sensing response, while all other SiC‐based systems display negative sensing responses. CO functionalization yields minimal sensing improvement in SiC systems, and its results are inferior to those of BC‐based sensors. BC‐based systems deliver excellent sensing performance across the board, making them favorable choices for sensor applications that demand high sensitivity. The strong positive response, particularly to H_2_S and C_2_H_4_, highlights BC’s favorable surface properties for gas‐molecule interactions.

Gas adsorption redistributes charge between the molecule and the nanocage surface, shifting frontier orbital energies and altering the HOMO–LUMO gap. Since conductivity depends exponentially on *E*_g_, even modest changes in *E*_g_ produce measurable conductivity responses. In B_16_C_16_ systems, chemisorption drives charge transfers up to 0.530 e (H_2_S_C4_BC), reducing the energy gap from 6.545 to 1.867 eV (%Δ*E*_g_ = −71.478% for H_2_S_C6_BC) and raising electrical conductivity from 2.12 × 10^12^ to 5.45 × 10^12^ S/m, with a sensing response of 1.569. Si_16_C_16_ systems show a different picture where physisorption keeps charge transfers below 0.279 e, gap changes under 4.183%, and sensing responses near zero, while recovery times fall to 10^−11^–10^−^^10^ s, presenting a clear advantage in reversibility.

## 4 Conclusion

In this study, the sensing potential of boron carbide (B_16_C_16_) and silicon carbide (Si_16_C_16_) nanocages was studied using B3LYP‐D3/6‐31G(d, p) functional of density functional theory (DFT). Three oil‐decomposed gases (C_2_H_4_, CO, H_2_S) were adsorbed on the nanocages to evaluate the sensor potential of these surfaces. Computational analysis of the adsorption potential of the nanocages was carried out using geometry optimization, adsorption energies, and electronic parameters, including FMOs, quantum descriptors, NBO analysis, and density of states. To further understand the sensing capabilities of designed systems, we studied weak interactions such as QTAIM and NCI. The sensor mechanism was evaluated for applying the studied systems as a sensor material. In all designed systems, the adsorption process causes a significant increase in bond lengths due to the movement of the electronic cloud. The highest adsorption energy values were calculated for BC systems compared with SiC systems. The adsorption of CO on BC nanocage results in the highest adsorption energy of −84.580 kcal in the CO_C6_BC system. Adsorption of C_2_H_4_ on BC causes the adsorption energy of −80.274 kcal in the C_2_H_4__C4_BC system. On the other hand, the SiC systems are less strongly bonded than BC systems, with the highest adsorption energy of −13.144 kcal in the H_2_S_C4_SiC system. FMO study reveals a decrease in the energy gap upon gas adsorption on BC nanocages. The least energy gap of 1.867 eV was computed for H_2_S_C6_BC and 1.914 eV for the H_2_S_C4_BC system prepared after the adsorption of H_2_S gas on BC nanocage. The remaining BC systems also show a significant reduction in the energy gap following the adsorption of C2H4 and CO. In contrast, SiC systems undergo an increase in energy gap apart from one system that has CO adsorbed on the SiC system. CO_C4_SiC is the only system with a reduced energy gap, at 2.960 eV, compared with 3.090 eV for the pristine SiC nanocage. Due to the decrease in the energy gap in BC systems, they exhibit better chemical reactivity, as predicted by quantum chemical descriptors. BC systems exhibit increased chemical softness and reduced chemical hardness, making them more reactive and enabling improved sensing performance. H_2_S functionalization on BC results in the highest chemical softness of 0.536 eV in H_2_S_C6_BC and 0.522 eV in H_2_S_C4_BC. Partial covalent and covalent bonds are the common nature of interactions between analytes and nanocages studied using NCI analysis. QTAIM analysis further confirms the nature of interactions, and all bonds are stable, as indicated by bond ellipticity values below 1. Results of adsorption and sensing experiments showed that H_2_S and C_2_H_4_ detection was improved by functionalizing BC‐based systems, whereas SiC‐based systems exhibited reduced sensitivity. Observational data indicate that materials derived from BC have higher electrical conductivity, making them more effective for sensing devices than SiC‐based systems. The BC systems exhibit high adsorption performance in terms of conductivity, sensitivity, charge transfer, and recovery time. Overall, the adsorption of oil‐decomposed gases (C_2_H_4_, CO, H_2_S) on BC nanocage produces better results than their adsorption on SiC nanocage.

## Funding

This study was supported by King Saud University, Riyadh, Saudi Arabia, through the Ongoing Research Funding Program (ORF‐2026‐645).

## Conflicts of Interest

The authors declare no conflicts of interest.

## Supporting information

Supplementary Material

## Data Availability

Data is available within the article and its supporting information.
