# Supplementary material for: Comparative Density Functional Theory Insights Into B16C16 and Si16C16 Nanocages for Sensing Oil‐Derived Fault Gases in Energy and Industrial Systems
Source: ChemistryOpen. 2026 Jun 16;15(6):e70245. doi: 10.1002/open.70245 (PMC13271869; doi:10.1002/open.70245)
Supplement: Supplementary file 1 — Supplementary Material [file OPEN-15-e70245-s001.pdf]

Supplementary Information

Comparative DFT insights into $B_{16}C_{16}$ and $Si_{16}C_{16}$ nanocages for sensing oil-derived fault gases in energy and industrial systems

Khalid Abdullah Alrashidi,[1] Hafiz Ali Rizwan,[2] Muhammad Usman Khan,[2]* Amir Sohail,[3]*

[1]*Department of Chemistry, College of Science, King Saud University, P.O. Box 2455, Riyadh 11451, Saudi Arabia*

[2]*Department of Chemistry, University of Okara, Okara-56300, Pakistan*

[3]*Department of Chemistry, College of Science, United Arab Emirates University, P.O. Box 15551, Al Ain, United Arab Emirates*

*** Corresponding author E-mail addresses:**

- **Dr. Muhammad Usman Khan**

  Email: usman.chemistry@gmail.com ; usmankhan@uo.edu.pk

- **Mr. Amir Sohail**

  Email: 201770097@uaeu.ac.ae

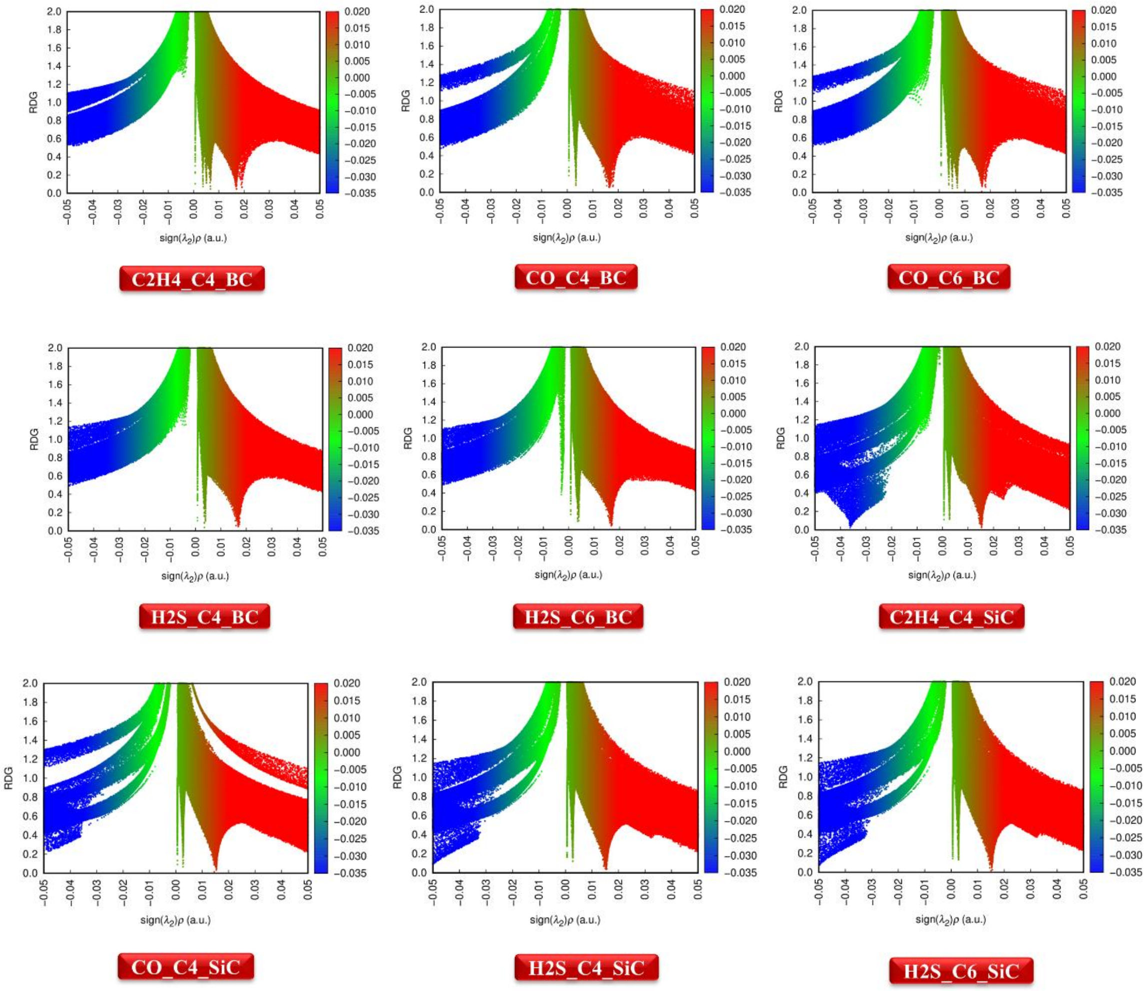

Figure S1. 2D-RDG graphs for the adsorption of C_2H_4, CO, and H_2S on SiC and BC nanocages.
